# Immersive ultraviolet disinfection of *E. coli* and MS2 phage on woven cotton textiles

**DOI:** 10.1038/s41598-022-17663-5

**Published:** 2022-08-02

**Authors:** Sean A. MacIsaac, Toni J. Mullin, Sebastian Munoz, C. Carolina Ontiveros, Graham A. Gagnon

**Affiliations:** Centre for Water Resources Studies, Department of Civil and Resource Engineering, Dalhousie University, 1360 Barrington St., Halifax, NS B3H 4R2 Canada

**Keywords:** Biomedical engineering, Civil engineering

## Abstract

Immersive ultraviolet disinfection provides a chemical-free technology for safer textiles, surfaces, and public spaces by inactivating communicable pathogens. This study examined immersive UV disinfection, using a disinfection cabinet, of *E. coli* and MS2 that was inoculated on white cotton T-shirts. The impact that porous materials have on UV disinfection is poorly understood with the majority of previous surface disinfection research focusing on hard, smooth surfaces. Several approaches were used in this study to characterize the light dynamics within the disinfection cabinet including colorimetric dosimetry coupons, biodosimetry, and spectroradiometry. Micro and macro geometry of porous surfaces are important factors to consider when using immersive UV technologies. The geometry of the cabinet impacted the distribution of emitted UV light within the disinfection cabinet and the physical properties of a porous material, such as the woven pattern of cotton, both contribute to UV disinfection efficiency. This work identified that light distribution is crucial for immersive UV technologies as the delivered fluence was highly variable within the disinfection cabinet and resulted in a difference of several logs of reduction for adjacent areas of T-shirt samples. Other inoculated areas achieved upwards of 1-log reductions values for MS2 and upwards of 2-log reductions for *E. coli*.

## Introduction

The interest and use of immersive UV-C technologies has increased dramatically as a response to the SARS-CoV-2 pandemic^1,2^. Immersive UV-C technologies use germicidal light to disinfect shared spaces or high-touch objects to reduce transmission of communicable illnesses. The SARS-CoV-2 pandemic exposed the need for safer shared spaces and the need for effective tools for reducing the viral load on high touch surfaces^3–6^. UV-C disinfection is well understood in the water industry for biological control and in healthcare settings for upper airway germicidal irradiation^7^. UV-C disinfection is considered an adjunct cleaning method to reduce the instance of hospital acquired infections (HAIs) via drug resistant organisms^8,9^. Donning and doffing of personal protective equipment (PPE) in healthcare settings has been identified as a viral vector in several studies^10–12^. UV-C disinfection of pathogen bearing clothing has the potential to reduce the instance of hospital acquired infections and can have uses for high traffic settings such as office buildings, stadiums, and university campuses^4,13^.

The dynamics of non-porous materials are well understood from a UV-C perspective whereas there exists a knowledge gap in applying immersive UV-C technologies to porous materials. One study investigated the efficacy of chemical disinfectants on porous and non-porous surfaces and demonstrated that textiles, such as cotton, were less efficient for disinfection by 2-log when compared to disinfection glass surfaces^14^. Understanding both the micro and macro-geometry of porous materials have significant implications for the efficacy of UV-C disinfection and must be considered when disinfecting complex surfaces^6,15–17^. For example, immersive UV-C technologies were used for emergency front facing respirator (FFR) reuse, where studies have shown FFR material type is instrumental in UV-C disinfection efficacy and governs the upper limit of achievable disinfection^1,6,17,18^. To the authors knowledge, there is no published study that investigates the efficacy of immersive UV-C disinfection on common, porous materials such as cotton. The importance of this knowledge gap is magnified when considering the growing interest to disinfect shared spaces, which consist of a mixture of porous and non-porous materials.

Disinfection cabinets are an immersive UV-C technology, which have been brought to market for several niche applications, such as clothing retailers, locker rooms, and laboratories. Disinfection cabinets provide 360° of UV-C exposure, which maximizes the illuminated area on porous objects and reduces the impact of shadowing. Another important factor to consider for these technologies is light distribution. Improper distribution of UV-C light can lead to inadequate disinfection of targeted objects. There are several tools that can be used to characterize the fluence delivered by immersive UV-C light sources such as UV-C dosimeter cards, biodosimetry, and spectroradiometry. Given the novelty of immersive UV-C devices, no standard exists for quantifying 360° fluences. This paper addresses this issue by characterizing a UV-C disinfection cabinet using common challenge microorganisms and radiometric techniques. Biodosimetry, chemical dosimetry (via dosimeter cards), and spectroradiometry were all used to characterize fluence using cotton T-shirts as the challenge garment. Cotton fabric is commonly found in clothing, furniture, and across all settings for UV-C applications and serves as a surrogate for porous materials. The outcomes of this study inform both the healthcare and UV-C industry regarding best practices when considering the disinfection of porous materials.

## Methods

### Disinfection cabinet setup

A commercially available UV-C disinfection cabinet equipped with eight, low pressure mercury halogen lamps were used for all validation studies in this manuscript^19^. Cycle times were programmed using the control panel on the cabinet that automatically controlled the irradiance period. A warmup cycle of was run on the cabinet prior to placing samples inside, which minimized the effects of lamp warmup to ensure a steady state of brightness during testing. Despite this practice, lamp brightness was not at a steady state for the first 20-s of use. Lamp warmup may increase the variability of disinfection efficiency for lower fluences. It is recommended that a warmup cycle is used in instances where the cabinet has been sitting idle for an extended time to mitigate variability in low fluence applications. The cabinet was also equipped with a locking mechanism to prevent inadvertent exposure to UV-C light during use. The characterization approaches used were designed to be minimally invasive to the overall function of the cabinet. For example, a thin USB cable was fed through the cabinet doors to power the spectroradiometer while keeping the cabinet seal intact.

Men’s medium, white, 100% cotton T-shirts were used for all samples and were taken directly from their package before use. This shirt type was chosen as it best represented a ‘baseline’ of cotton textile and is widely available for reproduction of this work.

### Microbial challenge test with MS2 and *E. coli*

**Figure 1:**
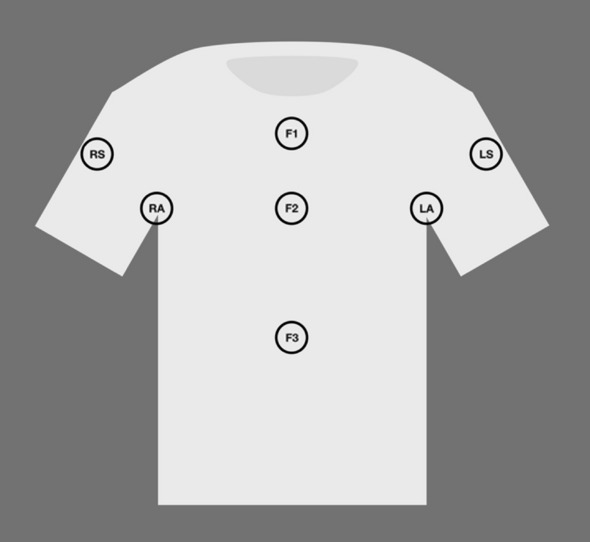
T-shirt inoculation regions for MS2 and *E. coli*. RS and RA refer to the Right Sleeve and Right Armpit; LS and LA refer to Left Sleeve and Left Armpit; F1, F2, and F3 refer to Front 1, 2, and 3.

MS2 and *E. coli* were chosen as challenge organisms due to their predicable response to UV-C light and were used for all microbial challenge organisms for all microbiological tests and were inoculated on garment target areas as shown in Fig. 1. MS2 bacteriophage and *E. coli* were added to target areas in six, 5 µL droplets within a 2 cm × 2 cm target area. Total inoculation volumes greater than 30 µL caused bleeding of fluids within the fabric that breached the 2 cm × 2 cm bounds. MS2 inoculated t-shirts were dried for 20 min on a stainless-steel table, and humidity was measured during experimentation using a DHT11 humidity sensor. *E. coli* inoculated shirts were not dried, as the *E. coli* inoculum were susceptible to cell death when drying occurred. Sterile petri dishes were placed between T-shirt layers during the inoculation and drying steps to ensure that the inoculum did not bleed through to the backside of the shirt. Petri dish dividers were removed before placing the garment in the UV-C cabinet.

**Figure 2:**
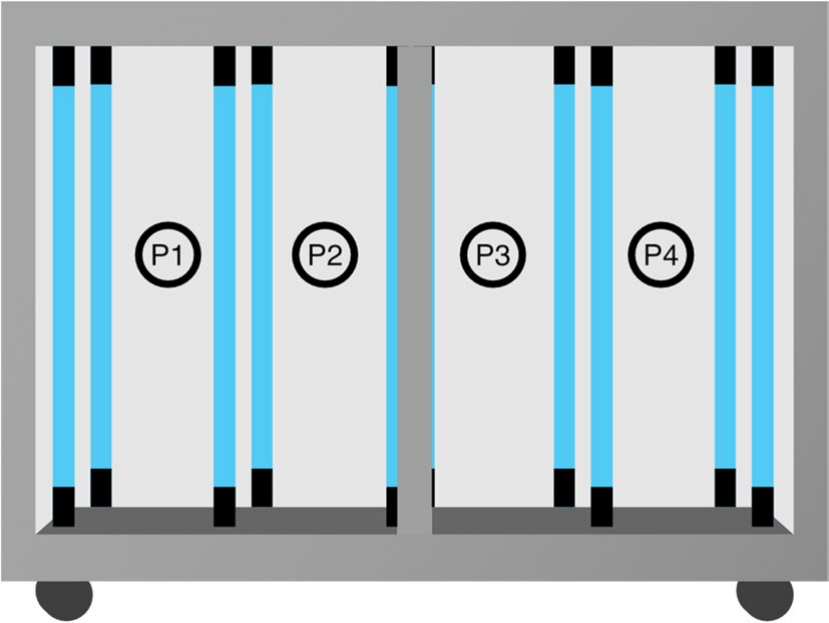
Positional indexing for hanging T-shirts within the UV-C disinfection cabinet.

White T-shirts were hung using a plastic hanger and placed inside the cabinet at a randomized location on one of the four positions within the cabinet. The left sleeve of each T-shirt was positioned nearest the cabinet doors in all experiments and the indexing for hanging positions is shown in Fig. 2. This orientation ensured that sampling locations were consistent in their relation to the UV-C lamps within the cabinet. The inoculation regions for T-shirts are depicted in Fig. 2. ‘R’, ‘F’, and ‘L’ refer to the right, front, and left side of the shirt respectively. ‘S’ and ‘A’ refer to the sleeve and armpit of the T-shirt. After UV-C exposure, coupons from the inoculated areas were cut with sterilized scissors and collected in a 50 ml Falcon tube and resuspended in phosphate buffer solution (PBS).

MS2 samples were enumerated using the double agar layer method with plate counts based on observed plaque-forming units (PFU) following EPA 1601 protocols^20^. *E. coli* was plated using Chromocult® Coliform agar (Millipore Sigma), a selective and differential chromogenic culture medium for the microbiological analysis of water samples. The use of this selective media provides robust quantification of UV-C treated samples while lowering the risk of sample cross-contamination, which is critical when working with non-sterile items such as cotton textiles. UV_254_ measurements were taken on a Hach DR5000 for control samples to account for the UV-C transmittance of the inoculum.

### UV-C emission validation with UV-C dosimetry

**Figure 3:**
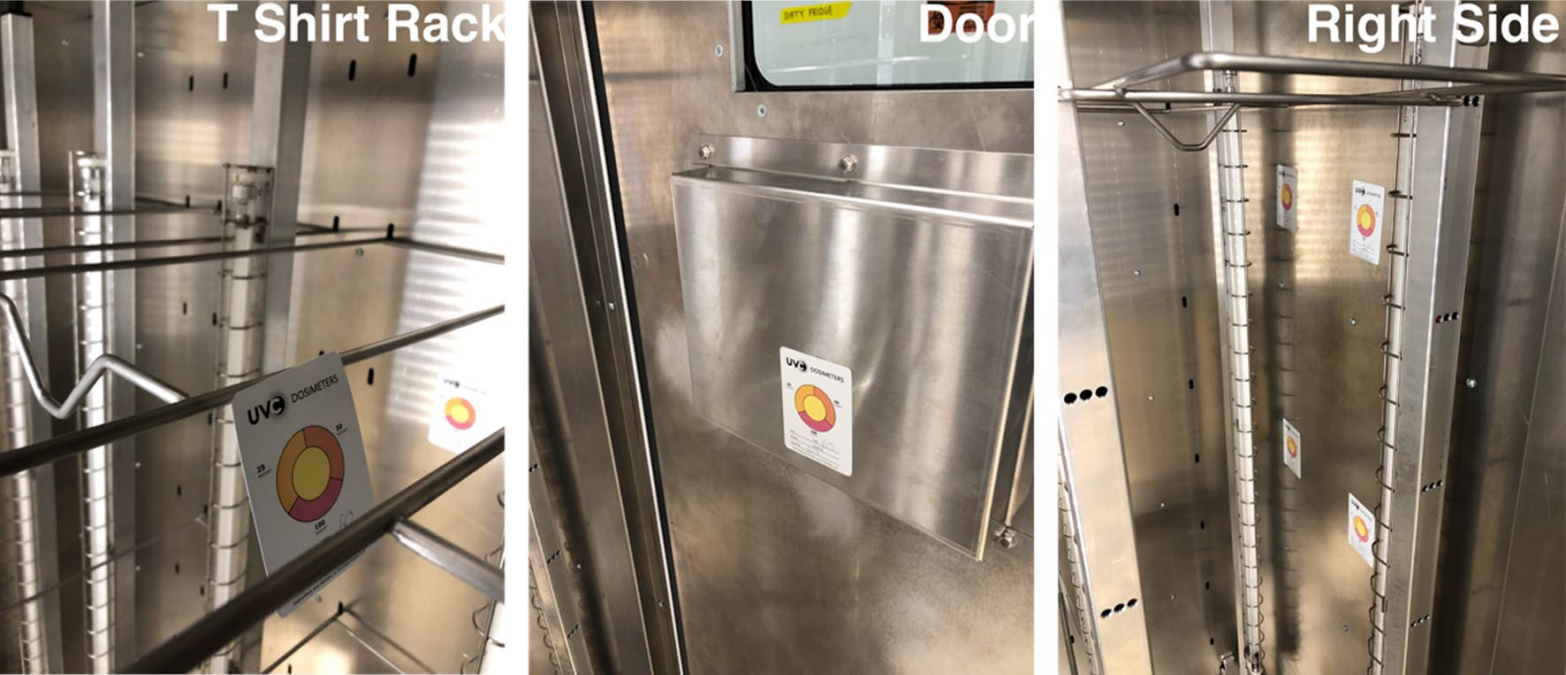
Internal layout of dosimetry coupons within the UV-C cabinet.

Dosimetry experiments were conducted in two phases to characterize a complete disinfection cycle. The dosimetry coupons used were colorimetrically sensitive to 25, 50, and 100 mJ cm^−2^ and provided an indication of the internal fluence achieved for all areas within the cabinet. The first phase consisted of a 30-s exposure time, and the second phase consisted of a 70 s exposure time. Exposure times were chosen based on the target use case of the device and also in consultation with the manufacturer of the cabinet^19^. The coupons have an adhesive back for attaching to surfaces. The coupons were placed in a 2 × 2 array spanning the dimensions of a hanging t-shirt on the inner lateral cabinet walls. The back wall coupon array covered the distance between the center of each UV-C lamp fixture. A coupon was attached on each cabinet door at the height of the centre of a hanging garment. Coupons were also attached on the racks in each hanging position within the cabinet. Figure 3 shows images of the dosimetry coupon configuration within the cabinet.

Photos of dosimeter coupons were taken immediately after UV-C exposure to mitigate fading of the indicator colour. Dosimetry coupons tended to fade to baseline colours if left sitting for times greater than four hours. Photos of UV-C exposed coupons were taken under consistent lighting conditions to control the impact of ambient light within the laboratory and were taken in the same location within the laboratory for each experimental run.

### Spectroradiometer measurement of UV-C fluence

**Figure 4:**
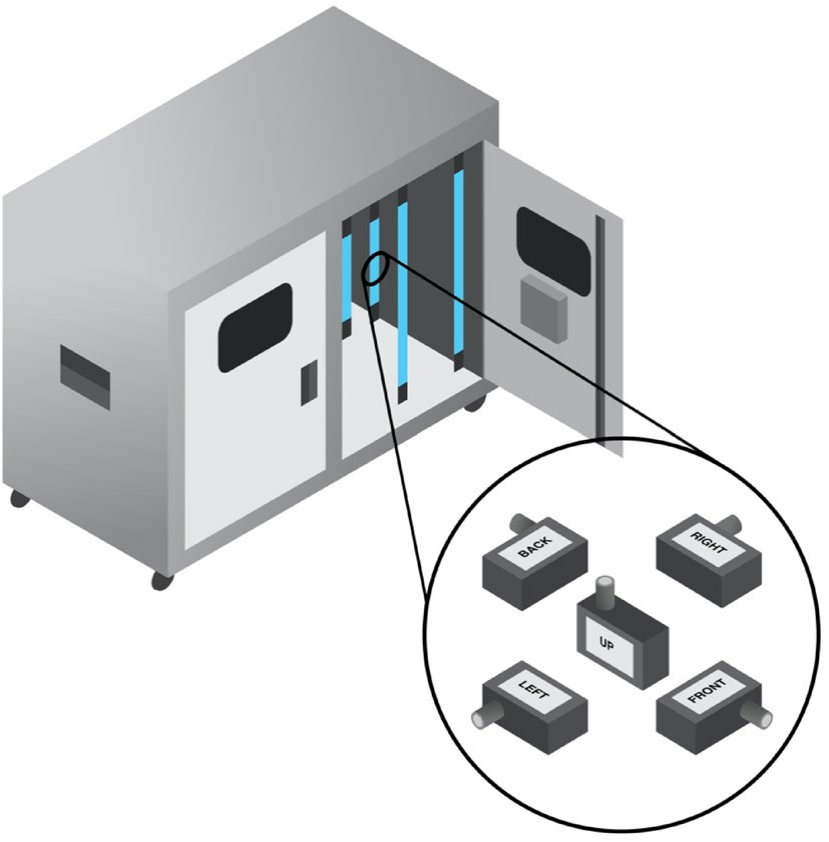
Orientation of spectroradiometer for irradiance measurements.

UV-C irradiance was measured using an OceanOptics USB4000 (OceanOptics, USA) spectroradiometer with a flat wire USB cable which enabled the radiometer to function in the cabinet while still maintaining a seal on cabinet doors. Spectroradiometer measurements were taken at each hanging position within the cabinet and were collected from five different orientations and are illustrated in Fig. 4. The fluence at each hanging location within the cabinet was then calculated by averaging the measured irradiance for each orientation of the spectroradiometer. Measurement of irradiance in multiple orientations at the same hanging position allows for an understanding of the distribution of light within an enclosed, UV-C illuminated volume.

### Investigation of UV-C light penetration through cotton layers

**Figure 5:**
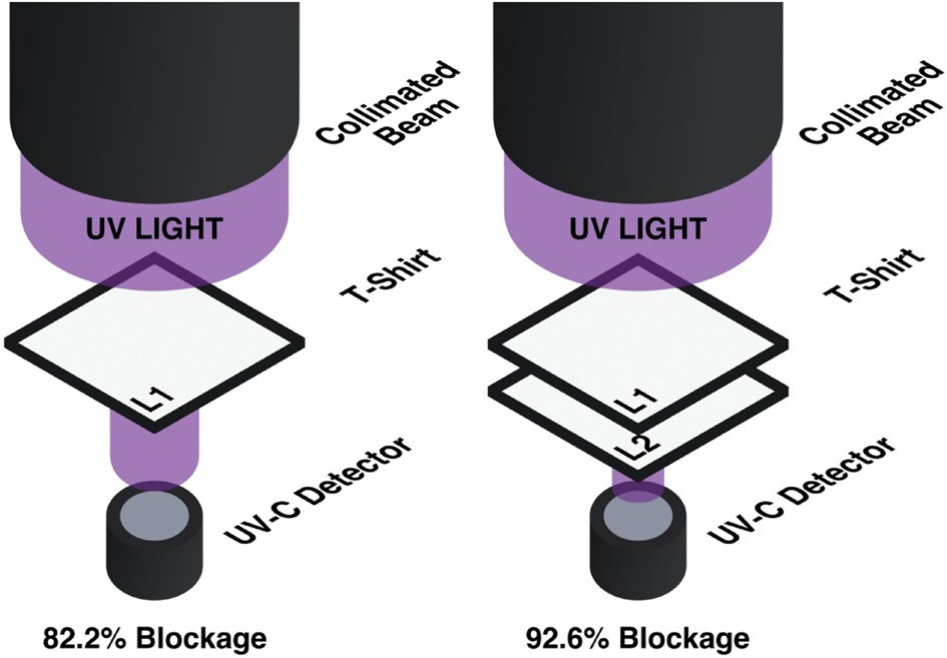
UV-C light penetration through different T-shirt cotton layers. L1 = one layer; L2 = two combined layers (n = 30).

The penetration of UV-C light through the T-shirt layers was investigated using a UV-C collimated beam. UV-C light penetration was tested on single (L1) and double layers (L2) of white cotton fabric using a collimated, 40 W low-pressure mercury lamp that was 25.5 cm from target textile. Collimated beams emit UV-C light in one direction whereas the UV-C cabinet provides 360° exposure of light. An Ocean Optics USB4000 spectroradiometer was used to measure light penetration to determine the proportion of UV-C light that is blocked by the layers of a cotton. Light penetration using a collimated beam is a surrogate for the disinfection cabinet as it mimics a conservative estimate for UV-C light blockage. The light penetration experiment consisted of L1 and L2 conditions. Cotton coupons were secured to the spectroradiometer detector surface which was then placed beneath the centre of the collimated beam and is shown in Fig. 5.

## Results and discussion

### UV-C fluence characterization

#### Dosimeter fluence characterization

Dosimetry revealed that the distribution of light within the disinfection cabinet is not uniform despite 360° emission of UV-C irradiation. Both 15 and 25 W lamps were used during the characterization process. Multiple wattages were tested due to supply chain constraints which could limit the availability of certain lamp models. The dosimetry results are summarized in Table 1. Dosimetry provides a colorimetric range specific to a UV-C fluence rather than a quantitative value. The 30-s irradiation cycle exposed all cabinet areas to a fluence of at least 43.8 mJ cm^−2^. The minimum delivered fluence for the shortest use case exceeds a 3-log reduction value for pathogenic viruses such as SARS-CoV-2 on hard surfaces^21^.

**Table 1:** Summary of light distribution within the UV-C disinfection cabinet for each lamp wattages.

	15 W		25 W	
	30 s	70 s	30 s	70 s
Left wall	100 (0)	100 (0)	90 (22.4)	100 (0)
T-shirt hanger	43.8 (12.5)	100 (0)	87.5 (25)	87.5 (25)
Right wall	100 (0)	100 (0)	90 (22.4)	100 (0)
Back wall	50 (0)	100 (0)	50 (0)	60 (22.4)
Door	50 (0)	100 (0)	50 (0)	50 (0)

Note that the minimum possible fluence for each colorimetric range is used for qualitative fluence values. Values in brackets represent standard deviation. (15 W n = 5, 25 W n = 4).

The 70-s exposure cycle resulted in the device achieving a minimum of 50 mJ cm^−2^ fluence in all cabinet areas. It should also be noted that several areas were very close to the 100 mJ cm^−2^ threshold of the dosimeters. Achieving an unobstructed fluence greater than those needed for a hard surface is key, as a porous surface reduces the delivered fluence due to shading, geometry, and textile characteristics^22^. The calculated fluence for the disinfection cabinet use-case exceed required fluence needed for a 3-log reduction of SARS-CoV-2^21,23,24^**.** Dosimetry coupons have an upper limit of detection and are qualitative in nature, which constrained their use as a characterization tool.

### Spectroradiometer fluence characterization

Spectroradiometer data indicated asymmetry within the disinfection cabinet across all orientations. Table 2 shows the fluence, given a 70-s cycle, averaged across hanging positions, and for each orientation of the radiometer. The Up orientation provided the lowest fluence, which was expected as no lamps are mounted to the cabinet ceiling. All UV light detected in the UP position reflected off the inner walls of the cabinet. A similar pattern is observed for the Front orientation as the majority of UV light detected from this position reflected off the cabinet doors. The distance from the ceiling to detect was approximately double the distance from the detector to the doors. Notably, the fluences for the Right and Front orientations of the detector were approximately half of the fluence for the Back and Left orientation. The cause of this discrepancy is unclear, as the layout of the cabinet is laterally symmetrical.

**Table 2:** Spectroradiometer fluence values for a 70 s cycle in each cabinet orientation. Values in brackets represent standard deviation.

Orientation	Average
Back	890 (90.1)
Front	436 (167)
Left	837 (231)
Right	426 (218)
Up	264 (7.51)

The results in Table 2 further highlight the limitations of dosimetry coupons. Every orientation of the spectroradiometer is above the 100 mJ cm^−2^ upper detection limit of the colorimetric coupon scale. This result indicates the proper consideration of dosimetry coupon drawbacks must be considered before use.

### UV-C light penetration through cotton layers

Light penetration using a collimated beam simulates light from a single source passing through a textile. An average irradiance of 3.97 W m^−2^ (n = 30) was measured by the spectroradiometer when no fabric was covering the detector. 82.1% and 93.0% of UV-C light was blocked when measuring the light penetration through L1 and L2 respectively.

#### UV-C cabinet light blockage via adjacent porous objects

Direct measurement of UV-C irradiance via spectroradiometry quantifies light intensity and addresses the sensitivity limitations of coupon dosimetry. The average delivered fluence is consistent across all hanging positions and is in excess of 100 mJ cm^−2^, but there are differences in the measured irradiance for specific orientations of the spectroradiometer. Lower fluences were calculated at all spectroradiometer positions where the detector was arranged in the Front and Up orientations as referenced in Fig. 5. Spectroradiometer data is in alignment with dosimetry data which showed similar patterns for this portion of the cabinet. Despite differences in irradiance, the experimental results indicate that light from each direction within the cabinet far exceed 100 mJ cm^−2^ in conditions where shadowing and textile micro-geometry are not factors. A detailed table containing this data can be found in “Supplementary Information”.

The impact of adjacent hanging garments was examined after determining the distribution of light for an empty cabinet. The spectroradiometer was hung from each of the internal positions withing the cabinet (P1, 2, 3, 4), while T-shirts were hung from each other position within the cabinet. The proportion of light blocked was then calculated and is summarized in Table 3. Positions 1–3 had similar light dynamics whereas P4 had a lower proportion of light blocked.

**Table 3:** Proportion of light blocked by hanging T-shirts at each location within the disinfection cabinet (± standard deviation).

Position	Percentage of light blocked
P1	41% ± 10%
P2	42% ± 7%
P3	40% ± 12%
P4	29% ± 12%

The light dynamics within the disinfection cabinet change as objects are placed inside. For example, a full cabinet (items hung at each position) would complicate the light dynamics within the irradiated volume. Characterization of both empty and full conditions provides insight regarding the amount of light that is blocked in each use case. Overall, the method outlined in this work to calculate a directional, average fluence provides a tool for understanding immersive UV-C devices.

### Microbial disinfection using MS2 and *E. coli*

MS2 and *E. coli* were used as challenge organisms to assess the disinfection cabinet disinfection capabilities. The relative room humidity was measured and ranged from 12 to 24%. The relative humidity may have impacted the recovery threshold for MS2 and *E. coli*. Dry air can lead to enhanced desiccation of viruses and bacteria on a garment before being treated within the cabinet.

#### Disinfection of cotton MS2 T-shirt coupons

**Figure 6:**
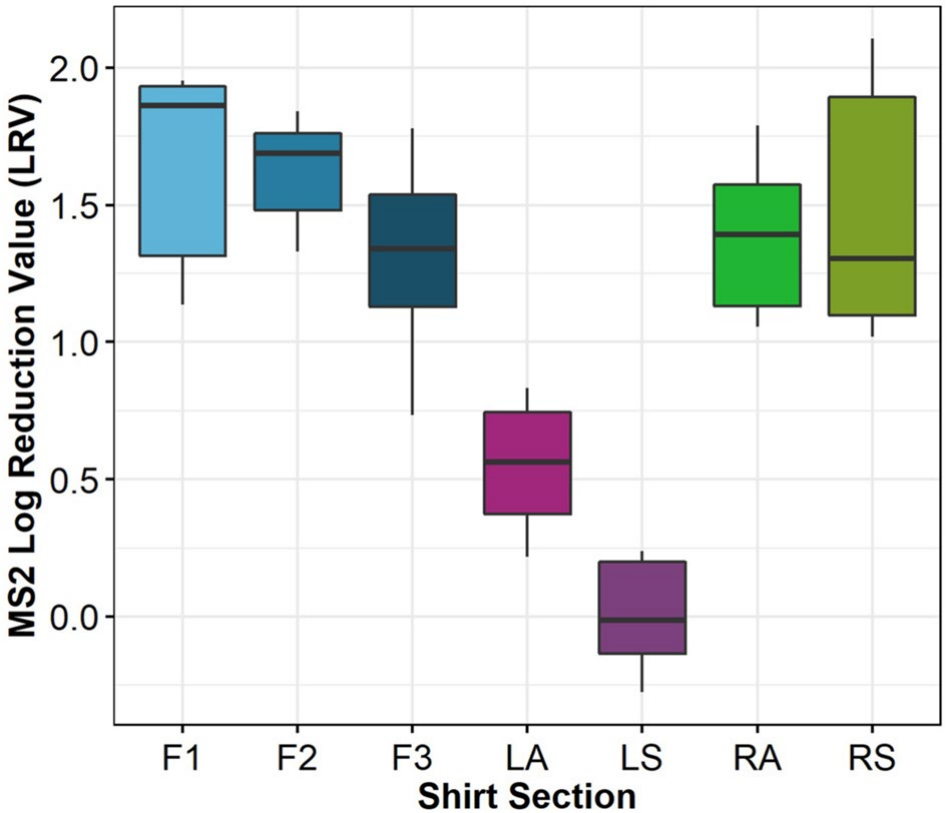
MS2 LRV for each inoculation location on hanging T-shirts (n = 3). RS and RA refer to the). Right Side (RS) and Right Arm (RA); Left Sleeve (LS) and Left Armpit (LA); Front 1,2,3 (F1, F2, and F3).

Log rection values for MS2 on each of the inoculated regions is provided in Fig. 6. MS2 control inoculum concentrations ranged from 7.5 to 8.5 PFU cm^−2^. Ultraviolet transmittance (UVT) of the inoculum was on average 72%. The recovery efficiency of MS2 was variable, with an average of 15.5% ± 23.5%.

The three front sections (F1, F2, F3) shown in Fig. 6 have average log reduction values (LRVs) of 1.58, 1.63 and 1.27 respectively. Furthermore, the RA and RS sections achieved 1.34 and 1.36 LRV. LA and LS locations had the lowest LRV with 0.47 for LA and − 0.07 for LS. A one-way ANOVA (α = 0.05) with shirt location as the factor and LRV value as the response confirmed there is a significant difference between shirt locations. A Tukey post-hoc test revealed that the front sections (F1, F2, F3) and the right sections (RA and RS) are not significantly different from each other (P-value > 0.05). Therefore, there is a significant difference between LA and LS sections and the rest of the locations tested.

#### Disinfection of cotton *E. coli* T-shirt coupons

*E. coli* was inoculated in the same manner as MS2; however, modifications to the protocol were implemented to compensate for difficulties in recovering *E. coli* from T-shirt coupons. The *E. coli* inoculation stock was adjusted to a concentration of approximately 10.5 log cm^−2^ to compensate for losses via cell death on dry cotton media. Additionally, *E. coli* had higher sensitivity to desiccation compared to MS2, which rendered the inoculum unrecoverable when dried. This led to the elimination of the 20-min drying step for *E. coli* experimentation. The UVT of the *E. coli* inoculum was virtually 0 due to the concentration needed for recovery. The recovery efficiency of *E. coli* resulted in an average of 4.0% ± 1.0.

**Figure 7:**
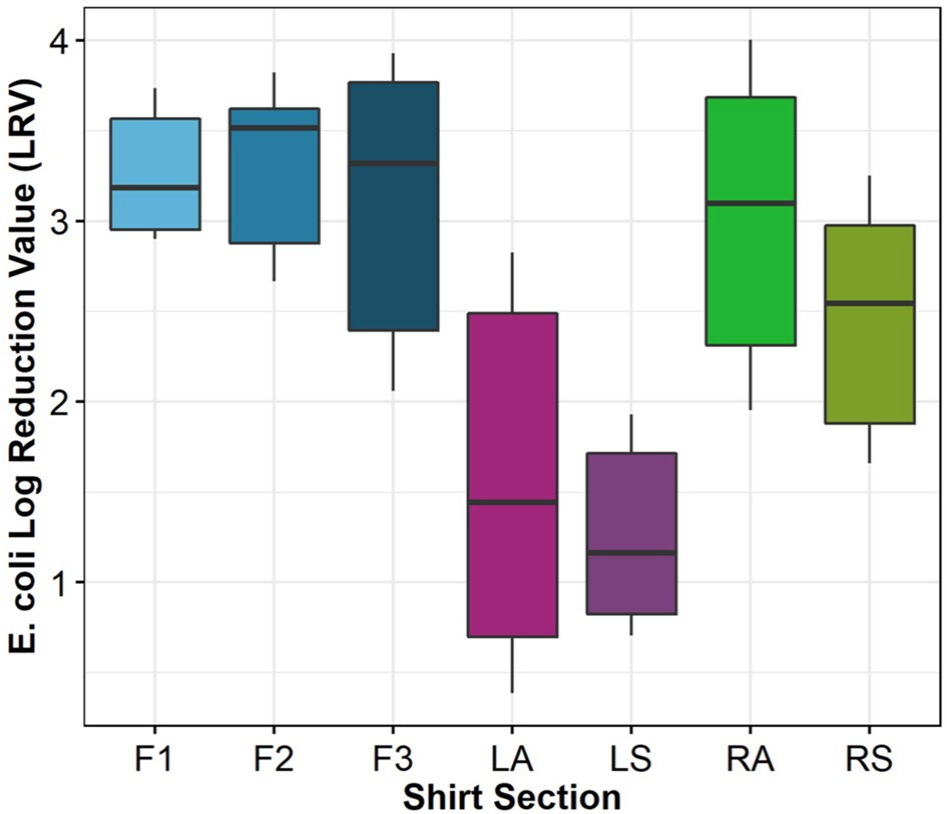
*E. coli* LRV for each inoculation location on hanging T-shirts (n = 3). Right Side (RS) and Right Arm (RA); Left Sleeve (LS) and Left Armpit (LA); Front 1,2,3 (F1, F2, and F3).

F1, F2 and F3 achieved higher average LRVs values of 3.1, 2.9 and 2.4 respectively, as shown in Fig. 7. Furthermore, the RA and RS sections achieved an average LRV 2.3 and 2.0. The LA and LS data showed the lowest LRVs of 0.7 and 0.9 on average. The LA section also resulted in the highest variability, with values ranging from 0.45 to 2.8 LRV.

One-way ANOVA (α = 0.05) with shirt location as the factor and LRV as the response revealed a significant difference between shirt locations. A Tukey Post-Hoc test revealed that sections F1, F2, F3 and RA were significantly different from LA, LS. Section RA was not significantly different from any of the sections. It is estimated that the lack of difference for this region is due to the variability in the data and not a physical phenomenon. The orientation of the LA and LS regions within the cabinet are hypothesized to be the root cause for the lack of disinfection in these areas compared to the right (RA and RS) and front (F1, F2 and F3) areas of the shirt.

#### Impacts on immersive UV-C disinfection

The physical layout of the disinfection cabinet is the primary source for significant differences in disinfection for T-shirt regions. Hanging racks within the cabinet are centered about the inner wall dimensions and not in relation to the arrangement of the UV-C lamps. The misalignment of light sources and hanging positions causes an uneven distribution of light within the cabinet. This work highlights the importance of understanding light distribution when designing and implementing immersive UV-C light technologies.

Material type also has an impact on disinfection efficiency in addition to light distribution. To the authors knowledge, there are no currently published manuscripts which examine the disinfection of textiles using immersive UV-C technologies. However, there is a large body of knowledge examining the use of UV disinfection technologies in water treatment. The differences between non-porous and porous surface disinfection are comparable to the disinfection of drinking water versus the disinfection of wastewater. UV-C is effective for both water matrices, but there are additional factors that must be considered for appropriate use. The same concept applies to non-porous surfaces versus porous surfaces.

As UV-based technologies are used in broader applications in areas such as schools, airports, or stadiums, the impacts that porous materials have on UV efficiency must be considered. This work provides baseline data which shows that fluences required to achieve disinfection on a porous surface are orders of magnitude greater than fluences need to disinfect a hard surface.

## Conclusions

A variety of methods for measuring fluence delivered by a UV-C disinfection cabinet were assessed in this study. In addition, the dynamics of light and the impacts of porous materials on UV-C disinfection were examined using biodosimetry. Complex, porous surfaces are poorly understood and are not typically used in UV-C research due to the inherent challenges in the macro and micro geometry of porous materials. The need for standard protocols when using immersive UV-C technologies on complex materials, objects, and spaces is apparent.

This study is also the first to demonstrate UV disinfection on cotton surfaces, which may have significant impacts on how disinfection technologies are used in the textile industry. The disinfection cabinet used in this work allows for immersive, 360° exposure to UV light for complex objects. The results of this study show that immersive UV technologies, when properly designed, can achieve 1-log to 3-log reductions on complex, porous textiles. This study also shows the importance of light distribution for immersive UV technologies. For example, the lack of disinfection on Left regions of sample T-shirts can be explained by the hanging rack position within the cabinet. A small error in design resulted in approximately a 1-log difference in disinfection efficiency when compared to other regions of the sample T-shirts. Misalignment in positioning caused areas of clothing nearest the cabinet doors to receive an asymmetrical amount of direct UV-C exposure compared to other regions.

Porous materials, such as cotton, should be considered in future work regarding the disinfection of surfaces and shared spaces. Understanding the disinfection dynamics of textiles and porous materials is necessary to properly integrate UV technologies in daily life. Ignoring factors that reduce disinfection efficiency, such as micro and macro geometry, that porous materials present can lead to insufficient disinfection. The authors suggest research into additional porous material types as material compositions also impacts UV-C disinfection efficiency.

## Supplementary Information

Supplementary Information 1.

## Data Availability

The data used in this study is available upon request by contacting the corresponding author.
