# Supplementary figures and images for: Immersive ultraviolet disinfection of E. coli and MS2 phage on woven cotton textiles
Source: Sci Rep. 2022 Aug 2;12:13260. doi: 10.1038/s41598-022-17663-5 (PMC9345007; doi:10.1038/s41598-022-17663-5)

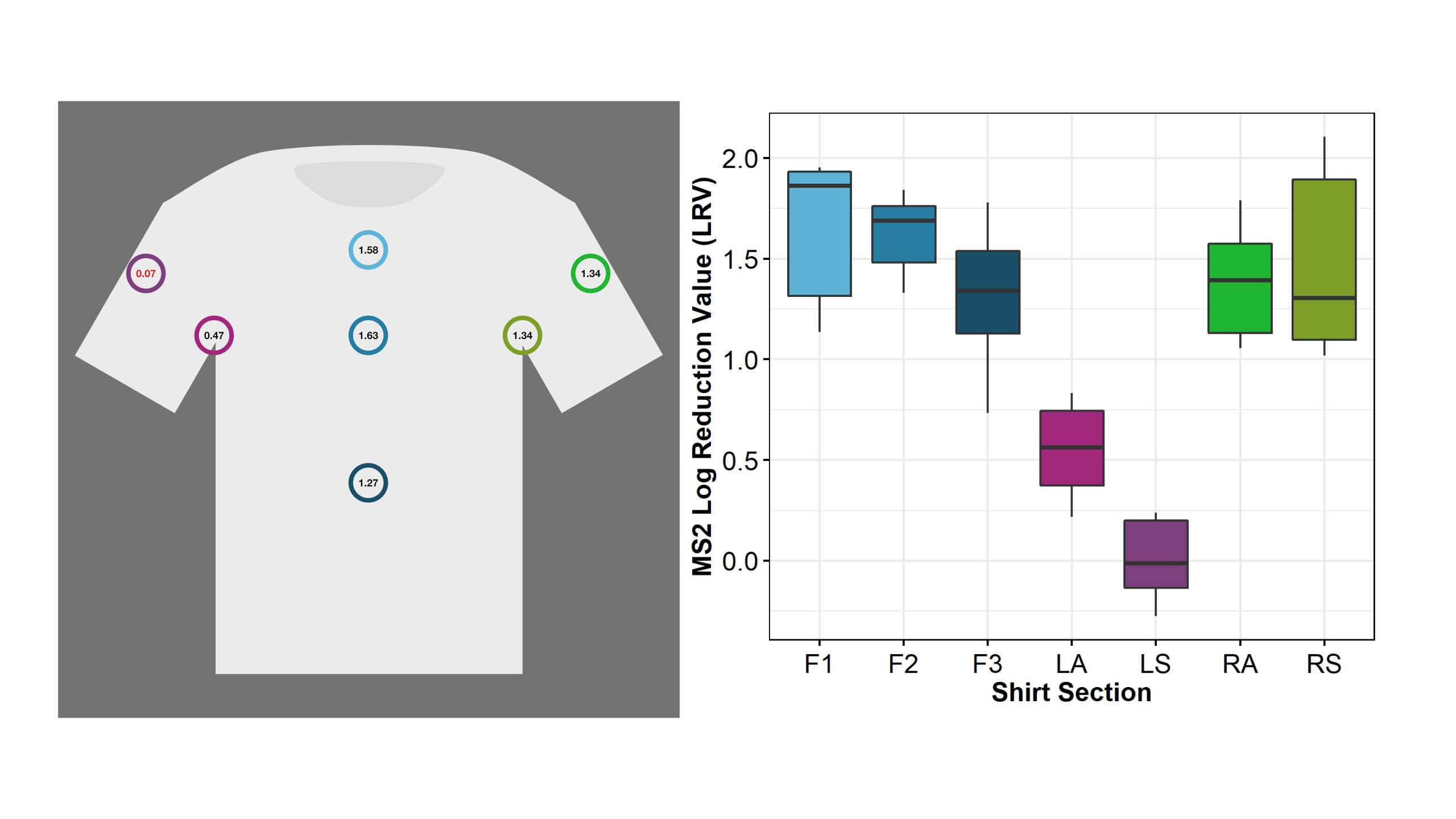

Supplement: Supplementary file 1 — Supplementary Information 1. [file 41598_2022_17663_MOESM1_ESM.png]
